# *Campylobacter jejuni* CsrA complements an *Escherichia coli csrA* mutation for the regulation of biofilm formation, motility and cellular morphology but not glycogen accumulation

**DOI:** 10.1186/1471-2180-12-233

**Published:** 2012-10-11

**Authors:** Joshua A Fields, Stuart A Thompson

**Affiliations:** 1 Department of Biochemistry and Molecular Biology, Georgia Health Sciences University, 1120 15th Street, Augusta, GA, 30912, USA

**Keywords:** *Campylobacter*, *Escherichia*, CsrA, Complementation, Glycogen, Motility, Biofilm, Morphology

## Abstract

**Background:**

Although *Campylobacter jejuni* is consistently ranked as one of the leading causes of bacterial diarrhea worldwide, the mechanisms by which *C. jejuni* causes disease and how they are regulated have yet to be clearly defined. The global regulator, CsrA, has been well characterized in several bacterial genera and is known to regulate a number of independent pathways via a post transcriptional mechanism, but remains relatively uncharacterized in the genus *Campylobacter*. Previously, we reported data illustrating the requirement for CsrA in several virulence related phenotypes of *C. jejuni* strain 81–176*,* indicating that the Csr pathway is important for *Campylobacter* pathogenesis.

**Results:**

We compared the *Escherichia coli* and *C. jejuni* orthologs of CsrA and characterized the ability of the *C. jejuni* CsrA protein to functionally complement an *E. coli csrA* mutant. Phylogenetic comparison of *E. coli* CsrA to orthologs from several pathogenic bacteria demonstrated variability in *C. jejuni* CsrA relative to the known RNA binding domains of *E. coli* CsrA and in several amino acids reported to be involved in *E. coli* CsrA-mediated gene regulation. When expressed in an *E. coli csrA* mutant, *C. jejuni* CsrA succeeded in recovering defects in motility, biofilm formation, and cellular morphology; however, it failed to return excess glycogen accumulation to wild type levels.

**Conclusions:**

These findings suggest that *C. jejuni* CsrA is capable of efficiently binding some *E. coli* CsrA binding sites, but not others, and provide insight into the biochemistry of *C. jejuni* CsrA.

## Background

Since its emergence as a pathogen of interest, *Campylobacter jejuni* has consistently been listed as one of the leading causes of infectious diarrhea throughout the developed world [1,2], and is the most common antecedent infection associated with onset of the neurological disorder, Guillain-Barré Syndrome [3]. Despite more than thirty years of rigorous investigation, the exact mechanisms by which *C. jejuni* causes disease in humans have eluded researchers. Publications of the genome sequences of various strains of *C. jejuni* revealed a surprising absence of many genes encoding proteins required for signal transduction and gene regulation. Although *C. jejuni* regulates gene expression in response to oxidative stress, iron availability, pH, and growth temperature, it does so with only three sigma factors, six sensor histidine kinases, and eleven response regulators [4-11]. This limited repertoire of regulatory elements and its overall lack of virulence factors has placed considerable importance on identifying novel mechanisms of pathogenesis and gene regulation to gain insight into the disease-causing mechanisms and capabilities of the organism in an effort to prevent and treat *C. jejuni* infections.

Observations reported by our laboratory and others have suggested an important role for the post-transcriptional regulator, CsrA (**C**arbon **s**torage **r**egulator), in the expression of several virulence-associated phenotypes in *C. jejuni*[12-15]. In other bacteria, CsrA (or its ortholog RsmA) is a small, regulatory protein capable of both activating and repressing the translation of mRNA into protein (reviewed by Romeo [16]). CsrA regulation is mediated by binding mRNA, often at or near the ribosome binding site (RBS), resulting in altered translation and stability of the message. CsrA, in turn, is regulated by the action of competitively binding two small, non-coding RNA molecules, *csrB* and *csrC*[16-20], whose expression is activated by the BarA/UvrY two-component system [19,21]. The CsrA pathway and the mechanism of regulation have been studied extensively in the γ-proteobacteria and further studies of the role of CsrA in various pathogens have extended its importance to the expression of virulence factors and the regulation of pathogenesis [22-26]. Despite these advances, very little is known about the mechanism of action of CsrA in the ε-proteobacteria. Examination of the *C. jejuni* genome [7,27-29] suggests that this bacterium lacks several genes in the CsrA pathway, including apparent orthologs of the small RNA molecules *csrB* and *csrC*[30], the *barA/uvrY* two-component signal transduction system, and *csrD* which is responsible for *csrB* and *csrC* turnover [31]. One report describing the role of CsrA in the gastric pathogen *Helicobacter pylori* indicated that CsrA was required for motility, survival under oxidative stress, and host colonization, and plays a role in the expression of several virulence and oxidative stress related proteins [23]. It was also suggested that the *H. pylori* ortholog was unable to function when exogenously expressed in *E. coli* because it failed to complement the glycogen accumulation phenotype of an *E. coli csrA* mutant [23].

Considering these observations in *H. pylori*, the phenotypes of a *C. jejuni csrA* mutant, and the lack of knowledge concerning the functions of CsrA within the ε-proteobacteria, we examined the ability of *C. jejuni* CsrA to complement the phenotypes of an *E. coli csrA* mutant with the hope of gaining further insight into the molecular mechanism of *C. jejuni* CsrA. Phylogenetic comparison revealed that *C. jejuni* CsrA exhibits variability in amino acids that constitute the published RNA binding domains, as well as in other residues that are important for CsrA-mediated regulation in *E. coli.* Surprisingly, although the *C. jejuni* ortholog was unable to complement the glycogen accumulation phenotype of *E. coli*, successful rescue of several other *E. coli* mutant phenotypes was achieved, demonstrating both similarities and differences in the *C. jejuni* and *E. coli* Csr systems.

## Methods

### Bacterial strains and routine growth conditions

All bacterial strains used in this study are listed in Table 1. Overnight cultures of *E. coli* strains were routinely carried out at 37°C on LB agar or in LB broth with shaking. One Shot® TOP10 chemically competent *E. coli* (Invitrogen, Carlsbad, CA) was used as a cloning host for TA-cloning procedures. *E. coli* MG1655 and TRMG1655 (*csrA::Kan)* were obtained from T. Romeo (University of Florida). When appropriate, *E. coli* strains were selected in LB medium using ampicillin (100 μg/ml) or kanamycin (50 μg/ml). Cloned genes were induced by the addition of 0.002% L-arabinose to the growth media. *C. jejuni* strain 81–176 was grown on MH agar at 42°C under microaerophilic contitions (10% CO_2_, 10% O_2_, and 80% N_2_) supplemented with 5% sheep’s blood (Remel, Lenexa, KS).

**Table 1:** Strains and plasmids used in this study

**Strain or plasmid**	**Description**	**Resistance**^**#**^	**Source or Reference**
Strains			
*C. jejuni* 81-176	Wild-type	Tet	[32]
*E. coli*			
MG1655	Wild-type		[33]
TRMG1655	*csrA*::*kan*	Kan	[33]
TOP10	Cloning host	Strep	Invitrogen
Plasmids			
pBAD-TOPO	Cloning vector containing *araBAD* promoter	Amp	Invitrogen
pBADcsrA^EC^	*E. coli csrA* cloned into pBAD-TOPO	Amp	This study
pBADcsrA^CJ^	*C. jejuni csrA* cloned into pBAD-TOPO	Amp	This study

^#^Tet, tetracycline; Kan, kanamycin; Strep, streptomycin; Amp, ampicillin.

### Phylogenetic analyses

**Figure 1:**
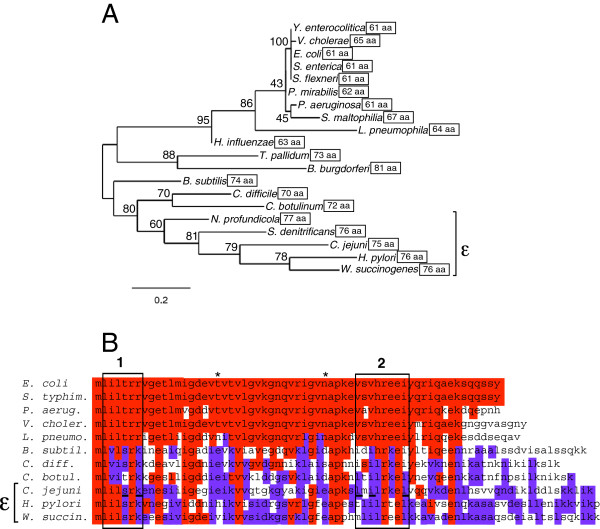
***C. jejuni *CsrA is divergent from the *E. coli *ortholog, including in the RNA binding domains.A**) CsrA orthologs from 20 diverse pathogenic and non-pathogenic bacterial species were aligned using CLUSTALW (neighbor joining). Numbers at tree nodes indicate the percent of bootstrap replicates that support the adjacent branches. Protein lengths (number of amino acids) are indicated to the right of each ortholog. Accession numbers for each protein are listed in Additional file 1: Table S1. **B**) Alignment of the amino acid sequences of CsrA orthologs. Regions 1 and 2 of *E. coli* CsrA important for RNA binding [35] are indicated by boxes and other amino acid residues important for CsrA regulation are indicated by an asterisk (*). Red shading indicates amino acids that are identical to those of *E. coli* CsrA; purple shading indicates amino acids that are different from *E. coli* CsrA but identical within the *C. jejuni*-containing clade of Figure 1A. Amino acids within RNA binding sequences 1 and 2 of *C. jejuni* CsrA that are conservative substitutions compared to *E. coli* CsrA are underlined.

Phylogenetic comparison of CsrA orthologs was performed by neighbor joining using CLUSTALW [34] within the VectorNTI 7.1 program suite (Invitrogen, Carlsbad, CA). Accession numbers for CsrA proteins used in the comparisons are listed in Additional file 1: Table S1. Bootstrapping (500 replicates) was performed to determine the statistical robustness of the clusters, and the percent of bootstraps that supported the clusters are indicated at each tree node (Figure 1A).

### DNA and protein techniques

Genomic DNA from *E. coli* and *C. jejuni* strains for use in PCR amplification was purified using the Generation Capture Column Kit (Qiagen, Chatsworth, CA). The plasmids used in this study were extracted and purified using the QIAprep Spin Miniprep Kit (Qiagen). PCR reactions were carried out using the Expand High Fidelity PCR System (Roche, Mannheim, Germany). Primers for PCR (Table 2) were synthesized by Integrated DNA Technologies (Coralville, IA). All DNA sequencing was performed by the GHSU Genomics Core Facility using an ABI Prism 337 XL DNA sequencer (Applied Biosystems, Foster City, CA). Western blots to validate the expression of CsrA^EC^ and CsrA^CJ^ were performed by using standard methods, with anti-his primary antibody (Penta-His Mouse Monoclonal, Qiagen; 1:1000 dilution) and goat, anti-mouse IgG-horseradish peroxidase secondary antibody (Pierce). All kits used in this study were used according to the manufacturer’s provided instructions.

**Table 2:** Primers used in this study

**Primer**	**Sequence (5**^**′**^**-3**^**′**^**)**
JAF 401	^#^GAG GAA TAA TAA ATG CTG ATT CTG ACT CGT CGA GTT GGT GAG
JAF 402	^*^TTA ATG ATG ATG ATG ATG ATG GTA ACT GGA CTG CTG GGA TTT
JAF 403	^#^GAG GAA TAA TAA TTA ATA TTA TCA AGA AAA GAA A
JAF 404	^*^TTA ATG ATG ATG ATG ATG ATG TTT GAT TAG TTT TTT GCT TA
	^#^Underlined nucleotides indicate a manufacturer recommended addition to remove vector encoded N-terminal leader sequence for expression of the native protein.
	^*^Underlined nucleotides indicate a manufacturer recommended addition to remove vector encoded C-terminal V5 epitope and add a polyhistidine tag for expression of the native protein.

### Plasmid construction

Plasmids used in this study (Table 1) were constructed using the pBAD-TOPO® TA Expression Kit (Invitrogen, Carlsbad, CA) and initially cloned into One Shot® TOP10 *E. coli*. The *E. coli* CsrA complementation plasmid, pBADcsrA^EC^, was constructed by amplifying the endogenous *E. coli csrA* allele from MG1655 genomic DNA using primers JAF401 and JAF402. The resulting amplicon was then TA cloned into the pBAD-TOPO vector such that CsrA expression was inducible by arabinose and detectable for western blot analysis by the addition of a C-terminal hexahistidine tag. The *C. jejuni* complementation vector, pBADcsrA^CJ^, was constructed by amplifying the *C. jejuni csrA* allele from 81–176 genomic DNA using primers JAF403 and JAF404 and cloning the resulting amplicon into pBAD-TOPO. Both complementation vectors and empty pBAD-TOPO plasmid were then transformed into the *E. coli csrA* mutant strain, TRMG1655, and recovered on LB agar containing ampicillin and kanamycin. In all phenotypic testing, we performed arabinose titration experiments (including samples without added arabinose) to determine the dose-responsiveness of CsrA expression and complementation ability.

### Glycogen accumulation

Glycogen accumulation was assessed using previously described methodologies [36]. Strains were grown at 37°C on Kornberg agar (1.10% K_2_HPO_4_, 0.85% KH_2_PO_4_, 0.6% yeast extract, 1.5% agar) with or without 2% (v:v) glycerol or 2% (w/v) sodium pyruvate; either glycerol or pyruvate was used as a carbon source as opposed to glucose due to the inhibitory effect of glucose on the *ara*BAD promoter [37]. Briefly, cultures were spotted on agar in the presence or absence of a carbon source and grown overnight at 37°C. Following incubation the cultures were stained by exposure to iodine vapor by inverting the plates over iodine crystals.

### Motility

Motility was quantitated as previously described [38], inoculating semi-solid LB agar (0.35% agar) by stabbing with an inoculating needle dipped into overnight cultures and incubating for 14 hours at 30°C in a humidified incubator. After incubation, the diameter of the zone of motility was measured. Experiments were performed a minimum of three times with no fewer than three replicates per experiment.

### Biofilm formation

Quantitative biofilm formation was assessed as previously described [33]. Briefly, overnight cultures were diluted 1:100 into LB broth. 100 μl aliquots were inoculated into a 96-well, round bottomed polystyrene microtiter plate and incubated statically at 26°C for 48 hours. Following incubation, biofilm accumulation was assessed by the addition of 25 μl of 1% crystal violet (in 95% ethanol) and incubating at room temperature for 15 minutes, followed by rinsing the wells three times with distilled H_2_O. Stained biofilms were quantitated by measuring the OD_570_ after solubilization in 80% DMSO for 24 hours at room temperature. Biofilm formation was also assessed qualitatively by aliquoting 1 ml of diluted culture into 5 ml polystyrene culture tubes and incubating statically at 26°C for 24 hours. Biofilms were then stained by the addition of 250 μl of crystal violet and incubated for 15 minutes, washed three times with distilled H_2_O, and photographed.

### Electron microscopy

Cellular morphology was assessed by scanning electron microscopy (SEM). Briefly, cultures were grown at 37°C for 18 hours in the presence or absence of arabinose. The cultures were then pelleted, and washed twice and resuspended in PBS (pH 7.2) and submitted to the GHSU Electron Microsocopy Core Facility for SEM. Twelve fields of view for each sample were randomly chosen for analysis and imaged at 10000x magnification. The resulting micrographs where then analyzed to determine the average length of the cells from each culture (n ≥ 150). Cells that were obviously undergoing cell division or those which were positioned on an inappropriate axis for assessing length were excluded from analysis. The resulting data were then analyzed by one-way analysis of variables (ANOVA) to assess statistical significance among the strains and to rule out variation within the twelve fields of view for each strain as a source of error.

### Statistical analysis

Results are presented as means ± standard error of means. Statistical significance was determined using ANOVA. *P* values of less than 0.05 were considered statistically significant.

## Results

C. jejuni *CsrA is evolutionarily divergent from* E. coli *CsrA and exhibits diversity in amino acid residues important for proper function in* E. coli.

Alignment of CsrA orthologs from a number of pathogenic and non-pathogenic bacteria (Figure 1A) showed that CsrA proteins of the ε-proteobacteria *C. jejuni* and *H. pylori* clustered distantly from most of the more thoroughly characterized enterobacterial orthologs. Furthermore, ε-proteobacterial CsrA proteins were of a larger size (75–76 amino acids) compared to those most closely related to *E. coli* (61–67 amino acids). The size difference was largely attributable to an C-terminal extension in the larger CsrA proteins (Figure 1B). In contrast to the high degree of amino acid conservation of CsrA orthologs of *E. coli*, *S. typhimurium*, *P. aeruginosa*, *V. cholerae*, and *L. pneumophila,* the CsrA proteins of *C. jejuni* and *H. pylori* exhibited substantial divergence from *E. coli* CsrA throughout (Figure 1B). This diversity was also apparent in two domains that were shown to be critical for RNA binding in *E. coli* CsrA [35]. In the most N-terminal RNA binding region (amino acids 2–7), *C. jejuni* CsrA shared four of six identical (two of six similar) amino acids with *E. coli* CsrA (Figure 1B). The C-terminal RNA binding region (amino acids 40–47) showed greater diversity, with only two of eight identical (three of eight similar) amino acids. Two additional amino acids that were shown to be important for regulation by *E. coli* CsrA (positions 19 and 35, marked by asterisks in Figure 1B) also were not conserved in *C. jejuni* CsrA. Together, these differences suggested the possibility that *C. jejuni* CsrA may regulate protein expression by binding to somewhat different RNA sequences than those bound by *E. coli* CsrA.

### C. jejuni *CsrA is unable to repress* E. coli *glycogen biosynthesis*

**Figure 2:**
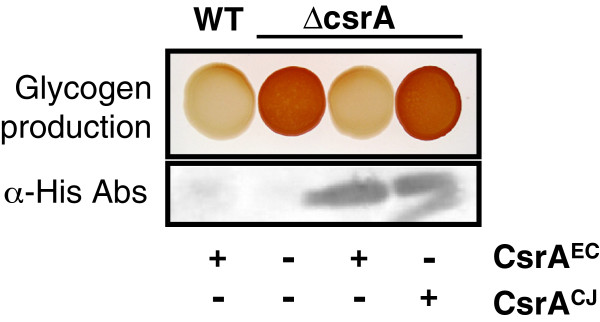
**Glycogen accumulation in wild type, *csrA *mutant, and complemented mutant strains of *E. coli. *** Top Panel) MG1655[pBAD], TRMG1655[pBAD], TRMG1655[pBADcsrA^EC^], and TRMG1655[pBADcsrA^CJ^] were spotted onto Kornberg agar supplemented with 2% glycerol and 0.002% L-arabinose and incubated at 37°C overnight. The following day, the strains were stained for glycogen accumulation by inverting over iodine crystals. Bottom Panel) Expression of his-tagged CsrA^EC^ and CsrA^CJ^ in TRMG1655 was confirmed by western blot using anti-his primary antibodies. Presence (+) or absence (−) of inducible CsrA^EC^ or CsrA^CJ^ in each strain is shown beneath the panels.

CsrA regulates *E. coli* glycogen biosynthesis via its effect on the genes in the *glgCAP* operon [12], and an *E. coli csrA* mutant accumulates significantly more glycogen than wild-type cells (Figure 2). A previous report indicated that the *H. pylori* ortholog of CsrA was unable to complement the glycogen biosynthesis phenotype of the *E. coli csrA* mutant [23]. Considering the close phylogenetic relationship between *C. jejuni* and *H. pylori,* we sought to determine the complementation potential of the *Campylobacter* ortholog for this phenotype. We expressed CsrA proteins from *C. jejuni* and *E. coli* (control) in an *E. coli csrA* mutant under the control of the arabinose-inducible *araBAD* promoter and examined glycogen accumulation on Kornberg agar in the presence of both glycerol (Figure 2) and pyruvate (data not shown). Glycerol and pyruvate were used as carbon sources to drive glycogen biosynthesis rather than glucose due to the inhibitory affect of glucose on the *araBAD* promoter [37]. In the presence of arabinose, we found that expression of *C. jejuni* CsrA in the *E. coli* mutant strain failed to repress gluconeogenesis, resulting in glycogen staining similar to that of the mutant strain harboring the vector alone. Expression of *E. coli* CsrA restored wild-type levels of glycogen staining, as expected, and the presence of the vector alone had no effect on glycogen accumulation in the wild-type strain. Expression of both orthologs of CsrA was confirmed by western blot analysis (Figure 2).

### *Expression of* C. jejuni *CsrA rescues the motility defect of an* E. coli csrA *mutant*

**Figure 3:**
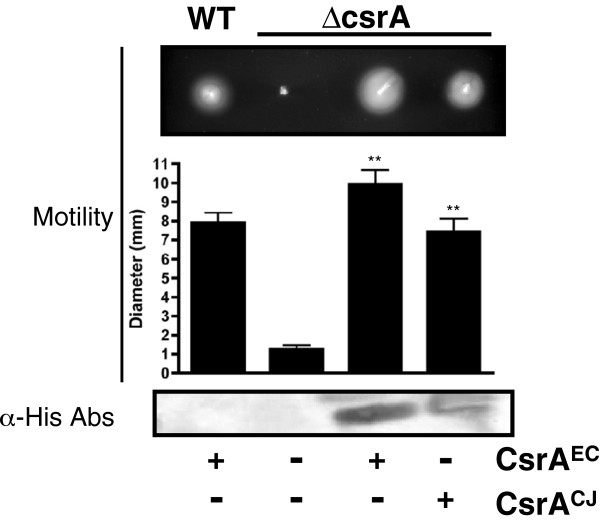
**CsrA**^**CJ **^**complements the motility defect of the *E. coli csrA *mutant.** The motility of MG1655[pBAD], TRMG1655[pBAD], TRMG1655[pBADcsrA^EC^], and TRMG1655[pBADcsrA^CJ^] was assessed on semisolid (0.35%) LB agar after 14 hours of growth at 30°C. Top Panel) Representative motility zones are shown, along with a graph of the measured zones of motility in three separate repetitions (n = 20/ repetition). Bottom Panel) Expression of his-tagged CsrA^EC^ and CsrA^CJ^ in TRMG1655 was confirmed by western blot using anti-his primary antibodies. Presence (+) or absence (−) of inducible CsrA^EC^ or CsrA^CJ^ in each strain is shown beneath the panels. ANOVA was performed to determine statistical significance of TRMG1655 expressing recombinant CsrA^EC^ or CsrA^CJ^ versus TRMG1655[pBAD] (** p<0.0001).

In *E. coli*, CsrA regulates motility by activating the regulatory operon *flhDC*[38], via stabilization of the *flhDC* transcript when post-transcriptionally bound by CsrA *in vivo*. In the absence of CsrA, *E. coli* cells exhibit a four-fold decrease in FlhDC expression resulting in a loss of motility. We compared the motility of wild-type and *csrA* mutant *E. coli* containing the vector alone to that of the *csrA* mutant strain expressing CsrA from *E. coli* or *C. jejuni* (Figure 3). We found that the *C. jejuni* ortholog significantly (p<0.0001) rescued the motility defect in a manner similar to that of *E. coli* CsrA (p<0.0001). Neither ortholog of CsrA successfully complemented motility in the absence of arabinose (data not shown) and the vector had no effect on motility in either the wild-type or mutant compared to the parent strains (data not shown). Western blots were used to confirm CsrA expression (Figure 3).

### C. jejuni *CsrA complements the biofilm formation phenotype of an* E. coli csrA *mutant*

**Figure 4:**
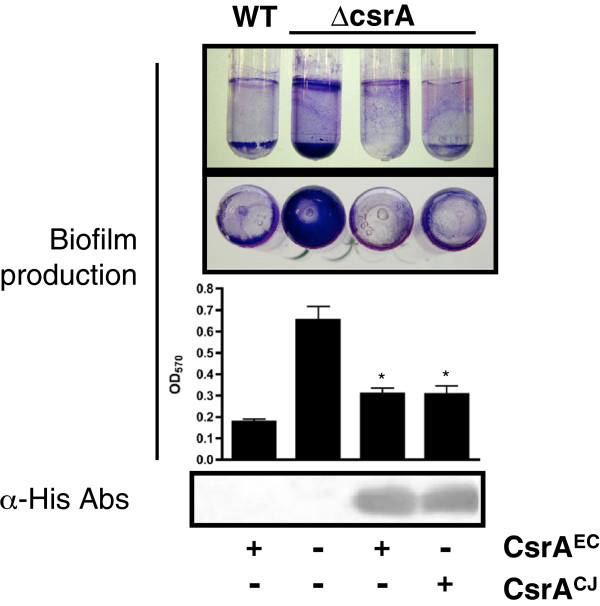
**CsrA**^**CJ **^**restores wild type *E. coli *biofilm formation.** Biofilm formation in MG1655[pBAD], TRMG1655[pBAD], TRMG1655[pBADcsrA^EC^], and TRMG1655[pBADcsrA^CJ^] were assessed in either polystyrene culture tubes (top panel; both side and bottom view of polystyrene culture tubes are represented.) or 96-well polystyrene microtiter dishes (quantitated in graph) using crystal violet staining after static growth for 48 hours at 26°C. Bottom Panel) Expression of his-tagged CsrA^EC^ and CsrA^CJ^ in TRMG1655 was confirmed by western blot using anti-his primary antibodies. Presence (+) or absence (−) of inducible CsrA^EC^ or CsrA^CJ^ in each strain is shown beneath the panels. ANOVA was performed to determine statistical significance of TRMG1655 expressing recombinant CsrA^EC^ or CsrA^CJ^ versus TRMG1655[pBAD] (* p<0.001).

Biofilm formation is repressed by CsrA in *E. coli,* resulting in the formation of excess biofilm by the *csrA* mutant. This phenotype is mediated by the effect of CsrA on the biofilm polysaccharide adhesin poly-*N*-acetylglucosamine (PGA) [15]. To determine the ability of *C.jejuni* CsrA to regulate biofilm formation in *E. coli,* we grew wild-type, mutant, and complemented strains statically, in 96-well polystyrene microtiter plates or in polystyrene culture tubes for 24 hours at 26°C and stained biofilms with crystal violet as previously described (Figure 4). As expected, the *E. coli csrA* mutant produced excess biofilm when compared to the wild-type; biofilm formation of neither the wild-type nor the mutant strains was affected by the presence of the vector (data not shown). As expected, *E. coli* CsrA complemented the mutant biofilm phenotype. Similarly, C*. jejuni* CsrA expression significantly reduced biofilm formation in the mutant to levels similar to that of wild-type (p<0.001). CsrA expression was confirmed by western blots (Figure 4).

### C. jejuni *CsrA expression restores the* E. coli csrA *mutant to wild-type morphology*

**Figure 5:**
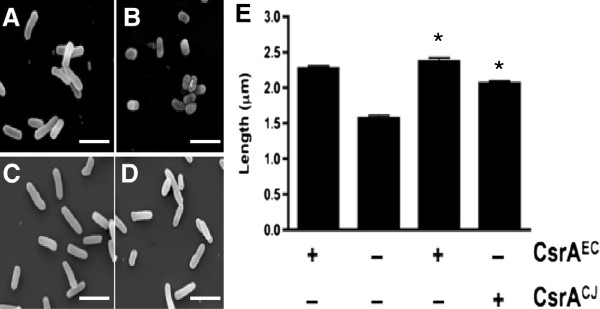
**CsrA**^**CJ **^**rescues the morphological phenotypes of the *E. coli csrA *mutant.** (**A**) MG1655[pBAD], (**B**) TRMG1655[pBAD], (**C**) TRMG1655[pBADcsrA^EC^], and (**D**) TRMG1655[pBADcsrA^CJ^] were grown overnight at 37°C in LB media supplemented with 0.002% L-arabinose and imaged by scanning electron microscopy. (**E**) Measured lengths of cells from SEM micrographs graphed for comparison. Presence (+) or absence (−) of CsrA^EC^ or CsrA^CJ^ in each strain is shown beneath the panels. ANOVA was performed to determine statistical significance of TRMG1655 expressing recombinant CsrA^EC^ or CsrA^CJ^ versus TRMG1655[pBAD] (* p<0.001).

We sought to examine reported morphological differences between the wild-type *E. coli* and *csrA* mutant strains and determine the capability of *C. jejuni* CsrA to complement the observed difference in cell size. We grew wild-type and mutant strains containing the vector alone and mutant strains containing the pBADcsrA^EC^ and pBADcsrA^CJ^ complementation vectors in the presence or absence of arabinose and measured the length of the cells (Figure 5). When grown in the absence of arabinose, we observed the reported elongated phenotype of the *csrA* mutant [36] which was unaffected by the presence of the vector. Interestingly, in the presence of arabinose, we observed a substantial increase in the length of wild type cells (Figure 5A), which was not evident in the mutant (Figure 5B; p<0.001). Expression of CsrA from both *E. coli* and *C. jejuni* (Figures 5C and 5D, respectively) significantly returned the mutant to the wild type dimensions (p<0.001). Western blot analysis confirmed expression of CsrA in the complemented mutant strains (data not shown).

## Discussion

Presently, studies from *C. jejuni* and the closely related gastric pathogen, *H. pylori*, report mostly the phenotypic effects of *csrA* mutation [13,23]. Furthermore, in *C. jejuni* as well as *H. pylori* the small RNA molecules (e.g. *csrB*, *csrC*) and the other proteins (e.g. CsrD) known to be involved in the Csr pathway in *E. coli* are either unidentified or absent [7,27-30,39]. This suggests that the molecular mechanisms may be somewhat different than those in *E. coli*. In this study, we sought to determine the capability of the *C. jejuni* CsrA ortholog to complement the phenotypes of an *E. coli csrA* mutant to gain insight into the mechanisms of *C. jejuni* CsrA function.

The *E. coli csrA* mutation has several phenotypes that can be used as tools for determining the capability of CsrA orthologs from other bacteria to complement the well-characterized *E. coli* strain. For instance, mutation of *csrA* in *E. coli* alters glycogen biosynthesis, biofilm accumulation, motility, and cellular morphology, as well as several other cellular processes. Mercante and colleagues [35] used the glycogen, biofilm, and motility phenotypes as a means to analyze the effects of comprehensive alanine-scanning mutagenesis of *E. coli* CsrA. In that study, the authors were able to identify which amino acids were most important for regulating glycogen biosynthesis, biofilm production, and motility, while also defining two regions of CsrA that are responsible for RNA binding.

When we compared representative CsrA orthologs from other bacteria, we found that *C. jejuni* CsrA is considerably divergent, as it clustered distantly from the *E. coli* ortholog. In part this is due to the significantly larger size of CsrA orthologs in the *C. jejuni* cluster (75–76 amino acids) as compared to the *E. coli* cluster (61–67 amino acids, Figure 1A). Considering the phylogenetic divergence of *C. jejuni* CsrA, we also examined the amino acid sequences of several CsrA orthologs of the pathogenic bacteria represented in Figure 1A to investigate the conservation of individual residues known to be important for the function of *E. coli* CsrA [35], and found that *C. jejuni* CsrA is considerably divergent in several key amino acid residues. Variability is found in both RNA binding domains, region 1 and region 2, although greater variation is found in region 2. The first region, residues 2–8, contains only two conservative substitutions (T5S and R7K) while the other four residues are identical. RNA binding region 2 is highly variable consisting of two residues that are identical to *E. coli* (R44 and E46), three similar amino acids (V40L, V42I, and I47L), and three non-conservative substitutions (S41M, H43L, and E45K). Between the defined binding regions, there were two non-conservative substitutions (T19E and N35E) we found to be particularly interesting because of their reported ability to improve the regulatory functions of CsrA in *E. coli*[35]. Presently, we are not able to draw any specific conclusions as to the significance of the individual amino acid substitutions in *C. jejuni* as compared to *E. coli*; however, it is likely that this divergence from *E. coli* plays a role in the ability of the *C. jejuni* ortholog to bind to *E. coli* targets appropriately.

In several studies, researchers characterizing the CsrA orthologues of different bacteria have used the glycogen biosynthesis phenotype of the *E. coli csrA* mutant to determine the functional similarities. One such study, of particular interest to our laboratory, reported that the *H. pylori* ortholog of CsrA would not functionally complement the *E. coli* mutant as it failed to repress glycogen biosynthesis [23]. It is likely that the *H. pylori* CsrA complementation failure was due to differences in the functional mechanism of ε-proteobacterial CsrA, however, this may have been specific to the two CsrA-binding sites of the *glgCAP* mRNA but not to other CsrA targets. To test this for *C. jejuni* CsrA, we examined the ability of CsrA^CJ^ to complement multiple *E. coli csrA* mutant phenotypes. We first expressed the *C. jejuni* ortholog in the *E. coli csrA* mutant and assessed its ability to repress glycogen biosynthesis under gluconeogenic conditions. Similar to *H. pylori* CsrA, the *C. jejuni* CsrA ortholog was incapable of repressing glycogen accumulation in the *E. coli csrA* mutant.

We next examined the ability of the *C. jejuni* protein to complement the motility, biofilm accumulation, and cellular morphology phenotypes of the *E. coli* mutant as well. As with glycogen biosynthesis, CsrA-mediated regulation of biofilm formation in *E. coli* is based on repression of a synthetic pathway, in this case the *pgaABCD* operon [15]. However, CsrA mediated expression of PgaABCD appears to be more complicated than that of glycogen biosynthesis, as it was reported that the mRNA leader sequence of the operon contains as many as six CsrA binding sites compared to the two binding sites observed on the *glg* leader sequence. Regardless of the complexity of the molecular mechanism of CsrA regulation of PGA we found that, when expressed in the *E. coli csrA* mutant, *C. jejuni* CsrA successfully complemented the biofilm formation phenotype (p<0.001).

Considering that the regulation of the *glg* and *pga* operons are both examples of CsrA-mediated repression of a biosynthetic pathway, we wanted to determine the ability of *C. jejuni* CsrA to substitute for its *E. coli* ortholog when the activation of gene expression is required. Wei and colleagues demonstrated that CsrA is a potent activator of *flhDC* expression and is therefore required for synthesis of the *E. coli* flagellum [38]. When we expressed *C. jejuni* CsrA within the non-motile *E. coli csrA* mutant the phenotype was completely rescued (p<0.001) suggesting that the *C. jejuni* ortholog is capable of promoting FlhDC expression.

Finally, we assessed the ability of *C. jejuni* CsrA to rescue an uncharacterized phenotype such as the altered cellular morphology of the *E. coli csrA* mutant. When CsrA was discovered, Romeo and colleagues reported that the *csrA* mutant displayed a greater cellular size as compared to the wild type, which was most obvious in early stationary phase [40]. This phenotype was explained as a possible indirect effect of endogenous glycogen accumulation. When we grew the wild type, *csrA* mutant, and complemented *E. coli* strains in LB media and observed their morphology via scanning electron microscopy, we observed a modest but significant decrease in the size of the mutant as compared to the wild-type as reported by Romeo [36] in the absence of arabinose; however, in the presence of arabinose we observed a marked decrease in the size of the mutant as compared to the wild type (p<0.001). This decrease in size was complemented by both orthologs of CsrA (p<0.001). As Romeo suggested that the size differences between the mutant and wild type may be due to the role of endogenous glycogen cellular morphology, it is possible that the presence of arabinose used for protein expression may play a separate metabolic role within the cell leading to the observed phenotype.

A number of studies have shown that regulation of mRNA targets by *E. coli* CsrA is complex [12,15,35,41]. Mercante et al. [41] showed that proper regulation depends on simultaneous binding of *E. coli* CsrA to multiple sites on target mRNAs, involving both of the RNA-binding surfaces of CsrA, using a multi-site bridging mechanism, and also the formation of higher order ribonucleoprotein complexes. Therefore, it is possible that the lack of regulation of the *E. coli glg* genes by *C. jejuni* CsrA is not due just to simple binding of one *glg* site vs. another, but rather due to changes in the dynamics (i.e. not ‘all or nothing’) of one or more of these bridging or ribonucleoprotein formation processes. For example, even moderately decreased affinity of *C. jejuni* CsrA for one of the *glg* sites may inhibit the formation of multi-site bridges and ribonucleoprotein complexes and therefore not result in productive regulation.

Finally, the binding of some but not all *E. coli* CsrA binding sites by *C. jejuni* CsrA infers that ε-proteobacterial CsrA binding sites are likely to show at least subtle differences from such sites in *E. coli*. It further underscores that predictive algorithms based solely or primarily on *E. coli* CsrA binding sites may be problematic for identifying CsrA binding sites in ε-proteobacteria and other divergent bacteria (Figure 1) [30], and that experimental approaches are preferable (such studies are ongoing in our lab).

## Conclusions

This study has shown that CsrA from the ε-proteobacteria *C. jejuni* exhibits substantial sequence divergence compared to previously studied CsrA regulators from other bacteria, including in the RNA-binding domains. The ability of *C. jejuni* CsrA to complement some, but not all, phenotypes of an *E. coli csrA* mutant demonstrates both conservation and divergence of function, and suggests that the *C. jejuni* ortholog may have differences in binding specificity relative to its *E. coli* counterpart. Studies to define the *C. jejuni* CsrA RNA binding site are ongoing.

## Competing interests

The authors have no financial or non-financial competing interests.

## Authors’ contributions

JAF participated in the study design, carried out all experiments in this work, and drafted the manuscript. SAT participated in the study design, performed phylogenetic analyses, and performed critical revisions of the manuscript. Both authors have read and approved the final manuscript.

## Supplementary Material

Additional file 1 **Table S1.** CsrA proteins used for phylogenetic analysis (Figure 1). Click here for file
