# Supplementary material for: Campylobacter jejuni CsrA complements an Escherichia coli csrA mutation for the regulation of biofilm formation, motility and cellular morphology but not glycogen accumulation
Source: BMC Microbiol. 2012 Oct 11;12:233. doi: 10.1186/1471-2180-12-233 (PMC3534301; doi:10.1186/1471-2180-12-233)
Supplement: Additional file 1 — Table S1. CsrA proteins used for phylogenetic analysis (Figure 1). [file 1471-2180-12-233-S1.pdf]

Table S1. CsrA proteins used for phylogenetic analysis (Figure 1).

**Species**	**Accession**
Yersinia enterocolitica	YP_001005173
Vibrio cholerae	NP_230199
Escherichia coli	NP_417176
Salmonella enterica	NP_461747
Shigella flexneri	NP_708504
Proteus mirabilis	YP_002150149
Pseudomonas aeruginosa	NP_249596
Stenotrophomonas maltophilia	YP_001971571
Legionella pneumophila	YP_123174
Haemophilus influenzae	NP_438973
Treponema pallidum	NP_219094
Borrelia burgdorferi	NP_212318
Bacillus subtilis	NP_391417
Clostridium difficile	YP_001086702
Clostridium botulinum	YP_001782343
Nautilia profundicola	YP_002606912
Sulfurimonas denitrificans	YP_392955
Campylobacter jejuni	YP_001000781
Helicobacter pylori	NP_224053
Wolinella succinogenes	NP_907087
